# Grating-assisted coupling to nanophotonic circuits in microcrystalline diamond thin films

**DOI:** 10.3762/bjnano.4.33

**Published:** 2013-05-07

**Authors:** Patrik Rath, Svetlana Khasminskaya, Christoph Nebel, Christoph Wild, Wolfram HP Pernice

**Affiliations:** 1 Institute of Nanotechnology, Karlsruhe Institute of Technology, 76344 Eggenstein-Leopoldshafen, Germany; 2 Fraunhofer Institute for Applied Solid State Physics, Tullastr. 72, 79108 Freiburg, Germany; 3 Diamond Materials, Tullastr. 72, 79108 Freiburg, Germany

**Keywords:** diamond devices, integrated optics, nanophotonics, waveguiding circuits

## Abstract

Synthetic diamond films can be prepared on a waferscale by using chemical vapour deposition (CVD) on suitable substrates such as silicon or silicon dioxide. While such films find a wealth of applications in thermal management, in X-ray and terahertz window design, and in gyrotron tubes and microwave transmission lines, their use for nanoscale optical components remains largely unexplored. Here we demonstrate that CVD diamond provides a high-quality template for realizing nanophotonic integrated optical circuits. Using efficient grating coupling devices prepared from partially etched diamond thin films, we investigate millimetre-sized optical circuits and achieve single-mode waveguiding at telecoms wavelengths. Our results pave the way towards broadband optical applications for sensing in harsh environments and visible photonic devices.

## Introduction

Integrated photonic circuits are of tremendous interest because they allow for realizing complex optical functionality in a compact and scalable fashion [1]. Alignment and stability issues commonly encountered in free-space setups can be avoided by moving to a chip-based architecture, in which photonic building blocks are laid out in much the same fashion as electronic integrated components. One of the most prominent materials for integrated optics is silicon, because of the availability of high-quality substrates, established fabrication routines, and a high refractive index [2–4]. For waveguiding in the telecommunications transmission window, thin silicon layers (surrounded by cladding material of lower refractive index) are required, which has led to the establishment of silicon-on-insulator (SOI) as a primary platform for nanophotonics [5–6]. However, silicon only provides a relatively small bandgap of 1.1 eV, which prevents waveguiding below 1100 nm. Furthermore, silicon is plagued be free-carrier absorption [7], which presents significant challenges for high-power applications and nonlinear optics. Some of these material shortcomings can be addressed by using chalcogenide-based devices for realizing tunable photonic circuits [8–10]. Alternatively, material systems with a wider bandgap are also actively pursued to extend the capabilities of photonic integrated circuits. Among the various options, group-III/IV nitride semiconductors, such as silicon nitride (≈5 eV), gallium nitride (3.37 eV) and aluminium nitride (AlN, 6.14 eV), have been investigated [11–13]. Because of their larger bandgap, these materials allow for waveguiding throughout the visible spectrum and do not suffer from free-carrier-based absorption effects or instabilities. While waveguiding at visible wavelengths is of importance for applications in biological sensing and spectroscopy, materials that also provide transparency in the long-wavelength range are equally sought after. In this respect CVD diamond has found a wealth of applications for the fabrication of windows that permit transmission in the long-IR or microwave regions [14]. In addition, diamond provides attractive material properties, such as biocompatibility, chemical inertness, high thermal conductivity, and mechanical hardness [15]. In addition, a large bandgap of 5.5 eV also makes diamond a prime candidate for the realization of optical components [16]. To date, the possibility of fabricating nanophotonic devices out of diamond has mainly been explored in single-crystalline substrates [17–22]. In order to make suitable waveguides, single-crystal diamond foils are transferred onto oxidized silicon carrier wafers and subsequently etched down to the target thickness of a few hundred nanometres. This elaborate procedure inherently limits the size of the available substrates (and thus also the later photonic devices) and provides limited device yield.

Here, in contrast, we employ direct deposition of CVD diamond thin films as a convenient alternative for realizing wafer-scale diamond substrates for integrated optics. Using microwave-enhanced CVD we prepare diamond-on-insulator templates up to six inches in diameter, without the need for further post-growth treatment or thinning. In microcrystalline films we demonstrate waveguiding over millimetre distances in the fibre-optics C-band. To characterize our platform, we design efficient grating couplers, which provide out-of-plane access to integrated photonic circuits with insertion loss of −5.0 dB. Our approach holds promise for transferring established silicon photonics technology to a diamond platform for applications in broadband optics and biological sensing.

## Results

### Fabrication of diamond photonic circuits

Diamond provides a relatively high refractive index of 2.4, which is well suited for tightly confining light in subwavelength structures [16]. In order to prevent radiative loss into the surrounding medium, the diamond waveguiding layer needs to be surrounded by a material of lower refractive index. Here in analogy to silicon-on-insulator (SOI) substrates we employ silicon dioxide as the lower buffer layer and air as the top cladding, to realize diamond-on-insulator (DOI) substrates [17,19]. Commercial, high-purity silicon wafers with atomically flat surfaces are thermally oxidized to a thickness of 2 μm. The resulting amorphous silica provides the later buried oxide and serves as the template for the CVD diamond overgrowth. The oxide thickness is chosen to provide optimal coupling efficiency for the grating structures described further below. A diamond nanoparticle seed layer is deposited onto the SiO_2_ film to initiate growth of polycrystalline diamond. We employ ultrasonification for 30 minutes in a water-based suspension of ultra-dispersed (0.1 wt %) diamond nanoparticles of typically 5–10 nm diameter to ensure uniform coverage of the underlying oxide surface [23]. Then the samples are rinsed with deionized water and methanol for cleaning and residual particle removal. After drying under nitrogen, the wafer is transferred into an ellipsoidal 915 MHz microwave plasma reactor [24]. Diamond films with a target thickness of 600 nm are grown at 1.8 kW microwave power. As feeding gas we employ a mixture containing 2% methane and 98% hydrogen at a base pressure of 80 mbar and a substrate temperature of 850 °C. Substrate rotation is applied to avoid angular nonuniformities arising from the gas flow. Growth rates are typically in the range of 1–2 µm/h. The diamond film thickness is controlled by timed growth combined with in situ interferometric measurement to allow for precise thickness monitoring. Because the growth rate is moderate and reproducible, the final film thickness can be controlled with high precision. After growth, the samples are cleaned in concentrated HNO_3_/H_2_SO_4_ to remove remaining surface contamination.

**Figure 1:**
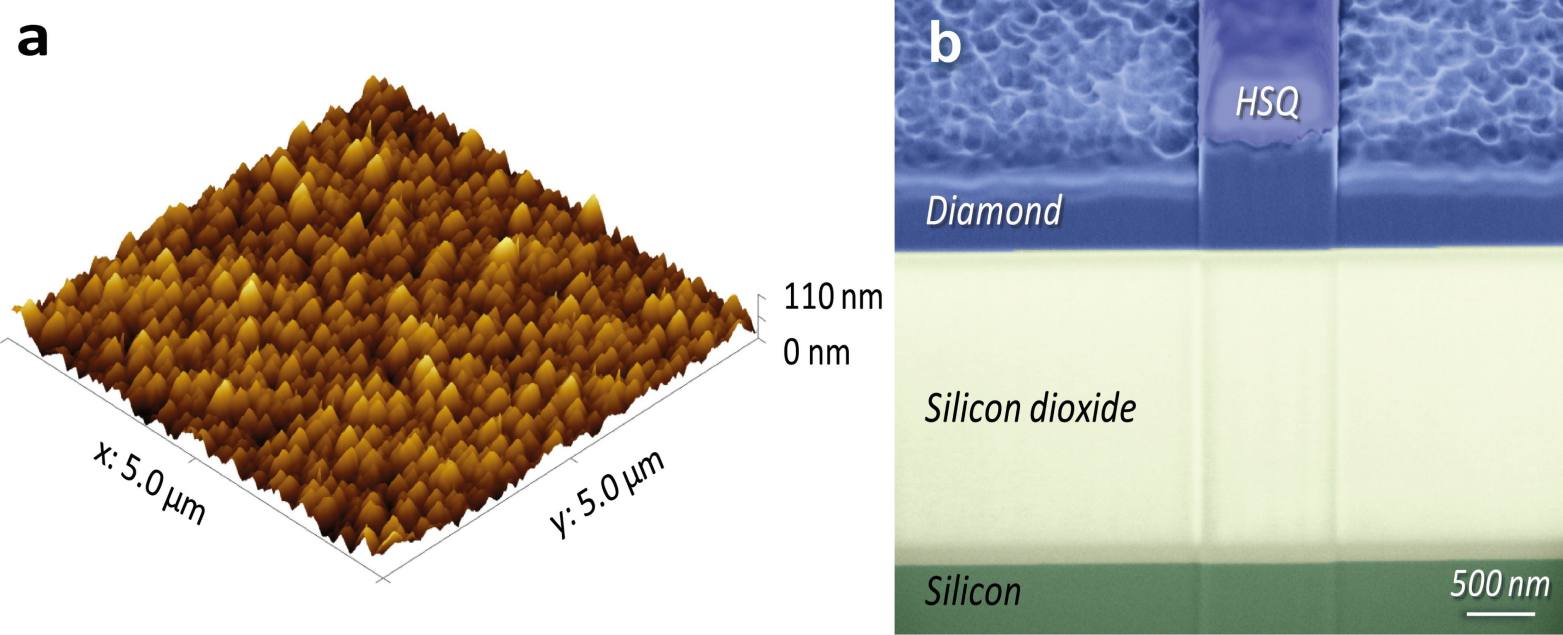
(a) AFM image of the as-grown surface of microcrystalline diamond-on-insulator substrates. Mean surface roughness of 15 nm rms is determined. (b) Cross-sectional SEM image of a nanophotonic waveguide cut by focussed-ion-beam milling. The diamond, e-beam resist, and buried oxide layers are marked in a false-colour overlay.

Following the film growth the resulting DOI substrates are inspected for thickness uniformity and surface roughness. We employ atomic force microscopy (AFM) and scanning electron microscopy (SEM) imaging to estimate the CVD diamond grain size and height distribution. A typical measured surface profile is shown in Figure 1a. From the AFM scan we obtain a mean roughness of 15 nm and a typical grain size on the order of 100 nm. The surface roughness leads to occasional diamond peaks that extend out of the surface. In order to structure the diamond layer for the creation of photonic devices, we therefore employ a thick photoresist, which uniformly covers the entire diamond surface and does not leave any peaks unprotected. Here we use the negative-tone electron beam (e-beam) lithography resist Fox15, which is spun onto the prepared wafers to a thickness of 400 nm. After exposure, Fox15 cross-links to a silica-like inorganic matrix, which provides good etching resistance as well as high spatial resolution during e-beam writing.

Photonic circuitry is designed and written into the Fox15 layer by using a JEOL 5300 50 kV e-beam system. After developing, the written structures are transferred into the diamond thin film by reactive ion etching (RIE) on an Oxford 80 system. We use oxygen/argon chemistry at high bias voltage in order to obtain highly anisotropic etching. Typical etch rates are around 25 nm/min, allowing us to precisely reach a desired etch depth. A false-colour SEM image of a typical ridge waveguide fabricated this way is shown in Figure 1b. Focussed ion beam (FIB) milling is used to cut through a waveguide cross-section, which is the reason for the line features at the edge of the waveguide. The FIB image reveals that the sidewalls resulting from the etching are near vertical, illustrating that the etch recipe is indeed highly anisotropic. Also visible in the image is the residual e-beam resist (labelled HSQ for Hydrogen silsesquioxane) on top of the waveguide.

### Design of focussing grating couplers

We fabricate nanophotonic waveguides with a width of 1000 nm using the procedure outlined above. Here we employ partially etched ridge waveguides as shown in the image in Figure 1b. By using such a waveguide geometry, the optical mode is confined more deeply into the diamond thin film compared to fully etched strip waveguides. This way, scattering effects due to the remaining surface roughness are reduced. Furthermore, we do not remove the Fox15 silica layer on top of the waveguide, which provides a further alleviation of scattering on the diamond top surface.

**Figure 2:**
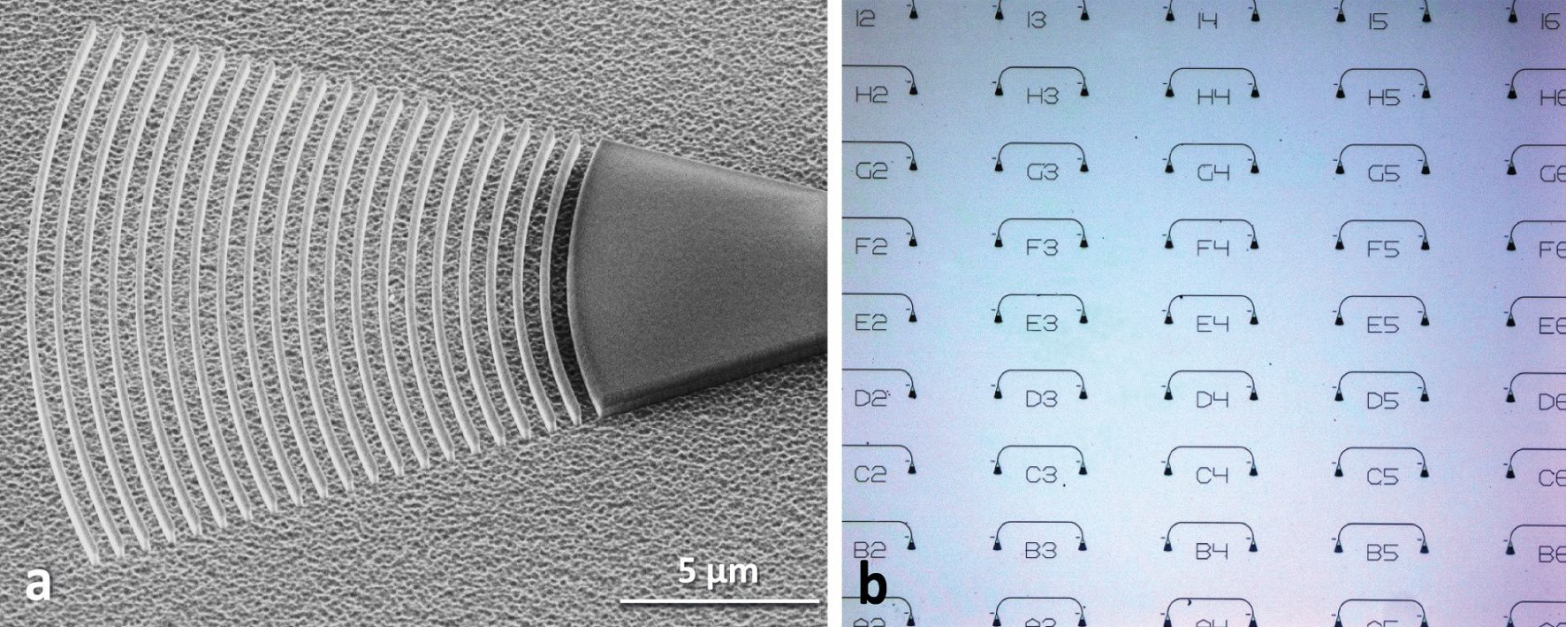
(a) SEM image of a fabricated focussing grating coupler. Light propagating through the incoming waveguide is scattered out of plane and collected by an optical fibre aligned to the grating section. (b) Optical microscope image of a fabricated chip containing several grating couplers connected by nanophotonic waveguides. The circuits enable characterization of the transmission profile of the grating couplers.

In order to access the optical properties of the ridge waveguides light needs to be transmitted through on-chip devices. While traditional butt-coupling using optical fibres aligned to cleaved facets of photonic chips is commonly employed [25], such an approach requires careful positioning of the input–output fibres with respect to the waveguide, which is time consuming and not suitable for the investigation of large numbers of devices. Therefore, we employ an alternative approach using grating couplers, which scatter light propagating inside the diamond waveguide out of plane [26]. As a result, scattered light can be collected from the top of the chip, which makes the assessment of many devices on a chip much easier. We use focussing grating couplers as shown in the SEM image in Figure 2a. The design consists of a Bragg grating, that scatters light to first order into and out of the waveguide and then focusses it into the onset of the waveguide. The coupler provides a coupling bandwidth of 50 nm centred around a mean wavelength that is determined by the grating period [27–28]. As shown in the optical micrograph in Figure 2b we employ two grating couplers connected by a nanophotonic waveguide in order to assess the transmission properties of the device. The circuit is aligned to two optical fibres bundled into a fibre array with a fixed separation between the fibre cores.

To measure transmission through the device, light from a tunable laser source covering the wavelength range from 1510 nm to 1620 nm (New Focus Venturi 6600) is coupled into the input fibre. After propagating through the photonic device and being collected with a second fibre at the output coupling port, the transmitted signal is recorded with a low-noise photoreceiver (New Focus 2011). The device is aligned against the fibre array using a computer-controlled motorized three-axis piezo stage.

**Figure 3:**
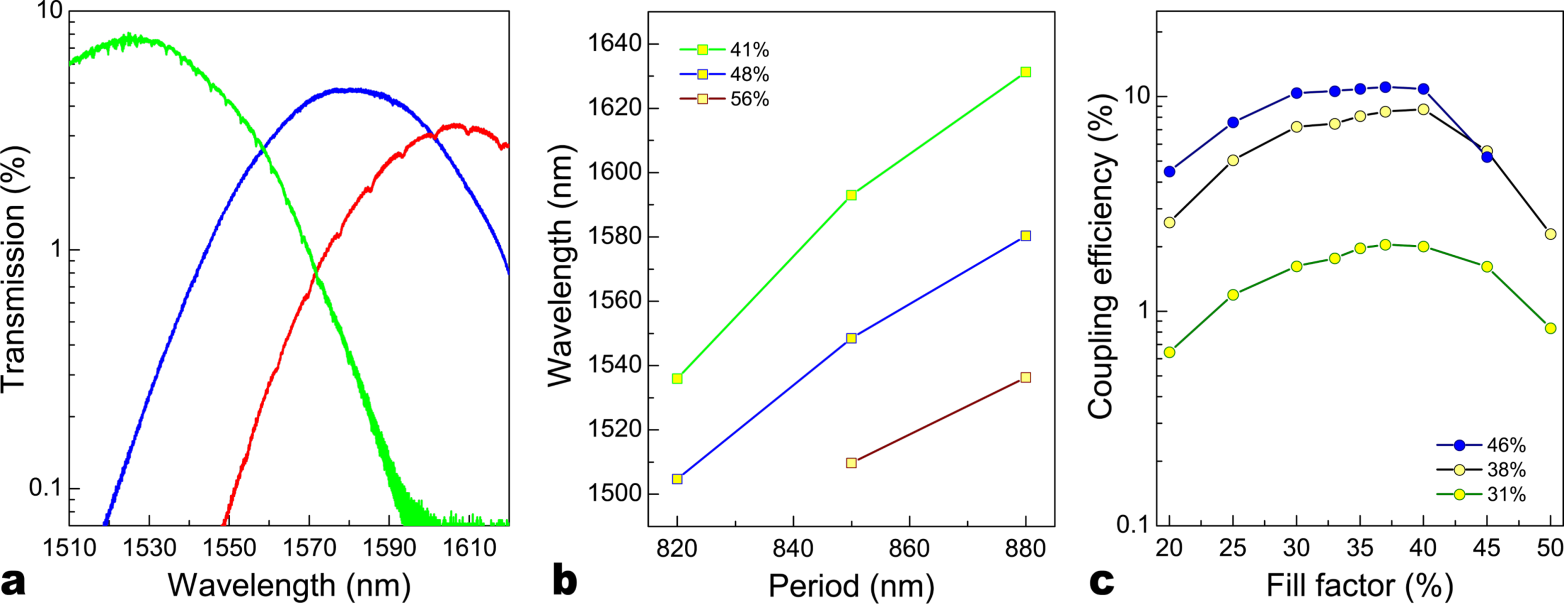
(a) Measured transmission spectrum of typical grating coupler devices. Best coupling loss at the central coupling wavelength of −5.0 dB is obtained. (b) The measured dependence of the central coupling wavelength on the grating period, which allows us to target specific wavelength windows over the coupler bandwidth of 50 nm. (c) The measured coupling efficiency for a pair of grating couplers in dependence of fill factor and etch depth. Optimal performance is obtained for gratings etched half way through the diamond layer.

Typical spectra recorded from devices as shown in Figure 2b are shown in Figure 3a. The coupler provides a 3 dB coupling bandwidth of 50 nm centred in the fibre-optics C-band at 1555 nm. For optimized grating couplers we find a best coupling efficiency of −5.0 dB, which is on par with or slightly better than comparable structures reported in SOI [29]. For use at different wavelengths the coupler bandwidth can be shifted by adjusting the period of the grating. As shown in Figure 3b, the central coupling wavelength varies almost linearly with increasing grating period. The data is obtained by fitting measured spectra from different circuits of varying period with a Gaussian profile to extract the central coupling wavelength.

The coupler efficiency is strongly dependent on the depth of the grating etched into the diamond thin film. Therefore we fabricate several rounds of devices that are etched to different depths in order to find optimal coupling performance. In Figure 3c we show measured results for devices with varying etch depth. The best coupling efficiency of −5.0 dB per coupler is found for devices that are etched half way into the diamond layer (300 nm deep in our case). For weakly etched gratings the maximum coupling efficiency measured in the tuning range of the laser increases up to a depth of 300 nm. Upon further etching, the coupling efficiency decreases, due to increasing modal mismatch with the intensity profile of the input-coupling fibre. However, for each etching depth the linear dependence of the central coupling wavelength on the grating period is maintained. Therefore we select partial etching down to 300 nm as the default structure for the following section.

### Measurement of long waveguide devices

Having established a robust fabrication and measurement approach for diamond nanophotonic circuits we fabricate devices containing long waveguides in order to estimate the propagation loss on-chip. We fabricate photonic circuits with a waveguide length up to 4.6 mm. To accurately measure the propagation loss through such waveguides we design a three-terminal photonic circuit. Light coupled into the input waveguide is split evenly by using a 50/50 Y-shaped beam splitter. Half of the light is then guided towards a reference-grating coupling port. The other half of the light propagates through the long waveguide and is then collected at a third output port. By measuring the transmission through both the reference port and the output port we can thus perform balanced detection of the properties of the long waveguide. By normalizing the output intensity to the reference intensity, the coupling loss occurring at the grating coupler can be eliminated. Taking into account the propagation length of the reference arm, we then obtain a nominal propagation loss of 5.3 dB/mm. This value is a factor of five smaller than the propagation loss reported in [30]. As obvious from the AFM image in Figure 1a, the measured loss is likely due to scattering at the surface due to roughness. Such scattering loss can be quantitatively estimated by using the Payne–Laycey model [31–32]. The scattering loss α (in units of dB/unit length) scales quadratically with the surface roughness σ as


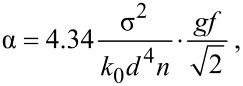


where *k*_0_ is the free space wave vector, 2*d* is the height of the waveguide and *n* the refractive index of the waveguiding layer. The factor 
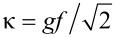
 consists of the analytical function *g*, which depends on the waveguide geometry and the function *f*, which depends on the index step of the waveguide and the correlation length of the surface roughness. Depending on the statistics used to describe the surface roughness, κ is bounded and an upper limit for α can be determined. For our waveguide geometry and the measured rms roughness of 15 nm the upper bound for scattering at the surface roughness is calculated as α ≤ 6.0 dB/mm for exponential statistics or α ≤ 9.4 dB/mm for Gaussian statistics, which is on the same order as the measured value for propagation loss in the diamond ridge waveguides. Since scattering loss is the dominant loss channel, in future work further improvement of the propagation loss will be possible by using surface polishing procedures to reduce the as-grown surface roughness.

## Discussion

Our implementation of wafer-scale diamond-on-insulator substrates offers new possibilities for nanophotonic integrated circuits. In contrast to existing opinions, we show that microcrystalline CVD diamond provides a viable platform for fabricating nanophotonic waveguides. Here we have demonstrated essential components for the investigation of optical functionality on chip, including efficient coupling ports, optical wire waveguides, and on-chip beam splitters. The possibility to deposit diamond thin films on high quality substrates with diameters of commercial relevance enables the design and layout of large photonic circuits on-chip. Because of the broadband transparency of diamond and the good thermal properties, such devices may also be driven at high optical input power. Thus, our approach holds promise for the realization of optical functionality that is currently not available in silicon technology. While our initial demonstration proves the viability of the concept, in future work advanced concepts from the nanophotonic community may also be ported to our diamond platform in order to make DOI a new addition to integrated optics.
